# Effects of Adipose-Derived Stem Cells and Platelet-Rich Plasma Exosomes on The Inductivity of Hair Dermal Papilla Cells

**DOI:** 10.22074/cellj.2021.7352

**Published:** 2021-10-30

**Authors:** Mohammad Ali Nilforoushzadeh, Nasser Aghdami, Ehsan Taghiabadi

**Affiliations:** 1 Skin and Stem Cell Research Center, Tehran University of Medical Sciences, Tehran, Iran; 2 Department of Regenerative Biomedicine, Cell Science Research Center, Royan Institute for Stem Cell Biology and Technology, ACECR, Tehran, Iran

**Keywords:** Adipose Stem Cells, Exosome, Hair Inductivity, Hair Loss, Platelet-Rich Plasma

## Abstract

**Objective:**

Hair loss is a prevalent medical problem in both men and women. Maintaining the hair inductivity potential of human dermal papilla cells (hDPCs) during cell culture is the main issue in hair follicle morphogenesis and regeneration. The present study was conducted to compare the effects of different concentrations of exosomes derived from human adipose stem cells (hASCs) and platelet-rich plasma (PRP) on the proliferation, migration and expression of alkaline pholphatase (ALP), versican, and smooth muscle alpha-actin (α-SMA) in human DPCs.

**Materials and Methods:**

In this experimental study, hDPCs, human hair DPCs and outer root sheet cells (ORSCs) were separated from healthy hair samples. The protocol of exosome isolation from PRP and hASCs comprises serial low speed centrifugation and ultracentrifugation. The effects of different concentrations of exosomes (25, 50, 100 µg/ ml) derived from hASCs and PRP on proliferation (MTS assay), migration (scratch test) and expression of ALP, versican and α-SMA (real time-polymerase chain reaction) in human DPCs were evaluated.

**Results:**

The flow cytometry analysis of specific cytoplasmic markers showed expression of versican (77%) and α-SMA (60.8%) in DPCs and K15 (73.2%) in ORSCs. According to NanoSight Dynamic Light Scattering, we found the majority of ASCs and PRP-exosomes (ASC-Exo and PRP-Exo) to be 30-150 nm in size. For 100 µg/ml of ASCs-Exo, the expressions of ALP, versican and α-SMA proteins increased by a factor of 1.2, 2 and 3, respectively, compared to the control group. The findings of our experiments illustrated that 100 µg/ml of ASCs-Exo compared to the same concentration of PRP-Exo significantly promote DPC proliferation and migration in culture.

**Conclusion:**

This study introduced the potential positive effect of ASC-Exo in increasing the proliferation and survival of DPCs, while maintaining their hair inductivity. Thus, ASCs-Exo possibly provide a new effective procedure for treatment of hair loss.

## Introduction

Hair loss is a common complaint of both male and female patients seeking beneficial treatments for this problem, worldwide (1) . As a general feature in human, hair plays a key role in beauty, social acceptance and self-esteem, therefore, hair loss is considered a major psychological challenge. Patent-pending statistics have shown increases in the costs of repairing hair loss over the past decade. Currently, hair loss is commonly treated with herbal extracts, platelet-rich plasma (PRP), adipose-derived stem cells (ASCs), keratinocyte-conditioned media and hair transplantation, none of which have yielded entirely satisfactory outcomes (2). Recent advances in tissue engineering and regenerative medicine have resulted in promising hair-loss treatments. Different research groups have so far made great efforts to develop the hair organoid structure in laboratories (3). 

Although many studies claim reconstructing the hair structure using murine stem cells and dermal papilla cells (DPCs), they have failed in human cells (4). The major causes preventing successful implementation of the results of animal models in human cells include the hair DPCs losing their trichogenic ability, an inadequate number of hair in people with severe hair loss and the lack of appropriate medical grade culture media (5, 6). As mesenchymal cells in hair follicles, the dermal papilla play the main role in regulating hair growth (7, 8). Preserving the potential hair inductivity of dermal sheath cells (DSCs) and DPCs in cell culture is the main factor in *in vitro* morphogenesis and regeneration of hair follicles (9). Using the currently available methods for cultivating human dermal papilla reduces the inductive capacity of the dermal papilla and the expression of specific dermal papilla biomarkers. Optimizing culture conditions for DPCs is therefore essential. The therapeutic capacity of different sources of exosomes, including mesenchymal stem cells (MSCs), has been recently assessed in regenerative medicine. Compared to other effective approaches in maintaining both *in vivo* and *in vitro* hair inductivity of DPCs, exosome-based treatments may be the most effective methods (10, 11). The advantages of using exosomes in experimental and clinical applications in hair loss include inducing endogenous mechanisms, simple processing, long-term storage, and reduced risks associated with immune responses (12). Research suggests that exosomes can induce cell proliferation, migration and angiogenesis, and promote tissue repair (13), nevertheless, the effects of exosomes on the trichogenic ability of DPCs and hair regrowth are yet to be defined. The present study was conducted to evaluate the effects of ASC exosomes (ASC-Exo) as well as PRP exosomes (PRP-Exo) on proliferation and migration of DPCs and the expression of hair-inductive genes. ASC-Exo and PRP-Exo contain different growth factors that are effective in cell proliferation. ASCs are easily accessible from various fat sources and improve the outcome for the treatment of hair loss. 

## Materials and Methods

### Isolation and culture of normal dermal papilla cells and outer root sheath cells from human scalp

In this experimental study, after obtaining informed consent from the donors, healthy hair samples were transferred to the cell culture laboratory of the Skin and Stem Cell Research Center of Tehran University of Medical Sciences. The Institutional Review Board and Ethical Committee of Tehran University of Medical Sciences (Tehran, Iran) approved this study (IR.TUMS. VCR.REC.1395.624). 

Human hair DPCs and ORSCs were isolated from healthy hair samples to investigate their potential for hair production. The cells were isolated and cultured as follows:

1. The hair samples were transferred to the laboratory in a Dulbecco’s Modified Eagle’s Medium (DMEM)/F-12 (1:1) (Thermo Fisher Scientific, USA) supplemented with 10% fetal bovine serum (FBS, Hyclone, USA)+ 50 μg/ ml of penicillin/streptomycin+50 µg/ml GlutaMAX™ Supplement (Thermo Fisher, USA).

2. The hair samples were rinsed in the Hanks’ Balanced Salt Solution (HBSS, Gibco, USA)+50 µg/ml of penicillin/ streptomycin (Gibco, USA), 70% ethanol and HBSS+50 µg/ml of penicillin/streptomycin, respectively.

3. The hair samples were placed in 1.2 unit/ml of dispase II (Gibco, USA) at 4ºC for 16 hours.

4. Part of the hair, i.e. its lowest one-third, was rinsed as dermal papilla in collagenase I (0.1%) (Sigma, USA) at 37ºC for 4 hours.

5. Collagenase I (Sigma, USA) was inactivated with a medium containing 10% FBS and the cells were passed through a 70-µm mesh filter and were then centrifuged at 1500 rpm for 5 minutes.

6. Part of the hair, i.e. its upper two-thirds, was rinsed as ORSCs in trypsin-EDTA (0.05%) (Gibco, USA) at 37ºC for 20 minutes.

7. Trypsin/EDTA enzyme was inactivated with a medium containing 10% FBS and the cells were passed through a 70-µm mesh filter and were then centrifuged at 200 rpm for 5 minutes.

8. Epithelial cells were cultured for 14 days in 6 well plates (TPP-Germany) coated with matrigel (Sigma, USA), and containing DMEM/F12 and gold KGM kits (Lonza, USA) at 37ºC in a humidified incubator with 5% CO_2_ . 

9. Dermal papilla cells were also cultivated in 6 well plates (TPP- Germany) with DMEM/F12 and +10% FBS+50 µg/ml penicillin/streptomycin+50 µg/ml Glutamax (Gibco, USA) at 37˚C in a humidified incubator with 5% CO_2_ . The cell culture medium was carefully changed every 3 days depending on the confluency of the cells.

### Flow cytometry for identifying dermal papilla cells and outer root sheath cells

The DPCs and ORSCs were collected and incubated with primary antibodies against α-SMA (R&D-USA), K15 (Abcam, USA), and versican (Abcam, USA) at 4ºC for 15 hours, and then incubated with secondary goat anti human-FITC (Abcam, USA) antibodies, goat anti human-FITC (Abcam, USA), After washing the collected cells with phosphate-buffered saline (PBS), the fluorescent cells were analyzed with flow cytometry (FC500; Beckman Coulter, Brea, CA, USA)

### Characterization of dermal papilla and hair outer root keratinocytes using immunohistochemical test

Immunohistochemical staining was performed according to standard protocols. In brief, dermal papilla and hair keratinocytes were fixed in 4% paraformadehyde solution and permeabilized using Triton X-100 (MERK, Germany, 0.2%). After washing with PBS/Tween (PBST) 0.05% (Sigma, USA), the cells were incubated in primary antibodies, including anti-versican (Abcam, USA), anti-α-SMA (R&D, USA) and anti-K15 (Abcam, USA), overnight at 4ºC. After washing, dermal papilla and hair keratinocytes were incubated in secondary antibodies for one hour at 37ºC. Specific cell markers were ultimately checked with fluorescence microscope Olympus Model DP71. DAPI was also applied for nuclear staining.

### Assessing alkaline phosphatase and toluidine blue staining in dermal papilla cells

The *in vitro* activity of alkaline pholphatase (ALP) in DPCs was investigated by culturing DPCs in 6 well plates and fixed in 4% paraformaldehyde solution and stained according to the ALP kit instructions (Sigma, USA). The extracellular matrix of human dermal papillae contains chondroitin 6-sulfate, heparan sulfate proteoglycans, laminin and type IV collagen. DPCs can be identified by determining the metachromatic features of these cells using toluidine blue staining. After fixation and washing with toluidine blue (Merck, Germany), the DPCs were stained and examined under a light microscope. 

### Isolating platelet-rich plasma from human cord blood

Approximately 200 ml of pooled human cord blood was collected in four 50 ml-conical tubes, containing 2×10^11^ total platelets on average. Pooled human cord blood was centrifuged at 300 g for 30 minutes at room temperature to obtain plasma. The plasma obtained from each tube was then transferred to a new 50 ml-conical tube and centrifuged at 200 g for 15 minutes. The PRP was ultimately stored at -80ºC for exosome isolation.

### Collection of conditioned medium from human adipose stem cells

According to the isolation procedure discussed, passage 3 hASCs were obtained from the Royan Stem Cell Bank in Tehran, Iran and cultured for 48 hours in DMEM/F12 medium supplemented with 10% exosome-free FBS and 1% penicillin/streptomycin in a humidified incubator with 5% CO_2_ at 37ºC before collecting the conditioned medium, which was then stored at -80ºC for exosome isolation.

### Exosome isolation from platelet-rich plasma and human adipose stem cells

The protocol of exosome isolation from PRP involves serial low speed centrifugation and ultracentrifugation. The hASCs conditioned medium and PRP were centrifuged at 300 g for 10 minutes to remove the cells. The dead cells were also removed by centrifuging at 2,000 g for 10 minutes. The supernatant was transferred to a 15-ml conical tube, and centrifuged at 10,000 g to remove cell debris. In the next step, the supernatant was ultra-centrifuged at 100,000 g for 2 hours (BECKMAN COULTER, MODEL: Optima L-100XP, USA) to obtain exosomes containing proteins. The supernatant was again collected in a new tube and centrifuged at 100,000 g for 2 hours at 4ºC. The pellet containing exosomes (40 µg/200 ml) was diluted in 1×PBS and stored at -80ºC for future use.

### Identifying the exosomes derived from human adipose stem cells and platelet-rich plasma

The concentrations of the exosomes derived from hASCs and PRP were specified using BCA protein assay kits (Thermo Fisher, USA). NanoSight LM10 (Nanosight) and Nanoparticle Tracking Analysis software version 2.2 were used to determine the particle size and distribution of the exosomes. Expressions of CD9, CD63, CD81, ITG101 and Calnexin proteins in the collected exosomes were analyzed using western blotting. The exosomes were morphologically characterized using a 100 kV-transmission electron microscopy (KYKYEM3200, Germany).

### Western blotting

The extracted proteins in the exosomes derived from hASCs and PRP were isolated using a 10% sodium dodecyl sulfate PAGE (SDS-PAGE) gel, and then transferred to a nitrocellulose membrane. The blots were blocked with 5% skimmed milk, then washed three times with a TBST buffer, and incubated at 4ºC for 15 hours with primary antibodies such as (anti-CD81, 1:100; Thermo Fisher Scientific, Waltham, MA, USA), (anti-CD9, 1:100; Thermo Fisher Scientific, Waltham, MA, USA), (monoclonal anti-TSG101, 1:100; Thermo Fisher Scientific, Waltham, MA, USA) , (anti-CD63, 1:100; Thermo Fisher Scientific, Waltham, MA, USA) and (anti-calnexin, 1:100; Thermo Fisher Scientific, Waltham, MA, USA). In the next step, the blots were detected by their corresponding secondary antibodies. Biorad’s ChemiDoc MP Imaging System was used for band detection.

### Analyzing the exosomes derived from human adipose stem cells and platelet-rich plasma with NanoSight dynamic light scattering 

The pellets of exosomes derived from hASCs and PRP were diluted in 100 μl of PBS, and the particle size and distribution of the exosomes were analyzed using NanoSight dynamic light scattering (632.8 nm laser) and BI 200SM Research Goniometer System (Brookhaven Instruments Corporation, Holtsville, NY, USA).

### Internalization of exosomes by dermal papilla

Whether the ASCs-Exo and PRP-Exo can enter DPCs was investigated. The procedure of labeling human dermal papilla is as described below: 

Centrifuging the ASCs-Exo was performed at 100,000 g for 60 minutes. Next, 200 µl of Diluent C of PKH26 kits (Sigma, USA) was added to the ASCs-Exo and PRP-Exo pellets. 200 µl of Diluent C was added to PBS- .

Then 200 µl dye solution in Diluent C was prepared by adding 4 µl of the PKH26 Dye solution to 200 µl of a Diluent C in a conical tube and mixing well. 200 µl of the ASCs-Exo and PRP-Exo (0.2 µg/µl) solution were added to 200 µl of the dye solution followed by performing pipette mixing. The ASCs-Exo and PRP-Exo solution and the dye solution were incubated for 5 minutes. The staining procedure was stopped by adding an equal volume of 1% bovine serum albumin (BSA) for 1 minute. Centrifuging The ASCs-Exo was centrifuged at 100,000 g for 60 minutes and was suspended in 200 µl of PBS- . The uptake of both ASCs-Exo and PRP-Exo was analyzed by dermal papilla with fluorescence microscopy

### Cell scratch assay

Human hair DPCs were seeded into 6-well plates with a density of 3×10^5^ per well of 6-well plates. Two days later, the plates were scratched with the tip of a 100 μl-sterile pipette. The serum-free cell culture medium containing 0, 25, 50 and 100 μg/ml of hASCs-Exo and PRP-Exo were added. Photos were taken from the scratched area and analyzed with ImageJ, and the width of the area was measured at time points of 0, 12 and 24 hours.

### Cell proliferation assay-MTS test

In this experimental study we analyzed the effects of exosomes on cell proliferation using the MTS assay. DPCs were cultured (10^4^ cells/well) in 96-well plates for 72 hours at 37ºC with different groups of exosomes. MTS assessments were performed using conditioned medium and the MTS solution (Promega, USA) at a 5:1 ratio. Absorbance was measured at 490 nm for each experimental group using an ELISA reader (Thermo, USA). 

### Scanning electron microscopy of human adipose stem cells and platelet-rich plasma-exosomes

The pellet of the exosomes derived from hASCs and PRP were suspended in 0.2-1 ml of DPBS. The samples were fixed for 12 hours in a 2.5% paraformaldehyde solution and a 0.1 M sodium phosphate buffer pH=7.2. One drop (5 μl) of the exosome suspension was added to clean slides, which were immersed in 1% osmium tetroxide for 1 hour, dehydrated in ethanol and distilled water, and then were dried under a ventilation hood. The samples were coated with gold in a sputter coater (Hitachi, Japan) before being examined through SEM (KYKYEM3200, Germany) with an accelerating voltage of 24 kV. The SEM images were analyzed in ImageJ to evaluate the exosomes’ size.

### Real-time polymerase chain reaction

Total RNA was extracted from cultivated dermal papilla with ASCs-Exo and PRP-Exo, using RNeasy Mini kits (Qiagen, Germany), and then reversed transcribed into complementary DNA (cDNA). Appropriate primers were designed for PCR as shown below:

*Versican*-

F: 5´TGAGCATGACTTCCGTTGGACTGA3´

R: 5´CCACTGGCCATTCTCATGCCAAAT3´

*α-SMA*-

F: 5´GTTTGAGACCTTCAATGTCCC3´

R: 5´CGATCTCACGCTCAGCAGTGA3´

*ALP*-

F: 5´CAACAGGGTAGATTTCTCTTGG3´

R: 5´GGTCAGATCCAGAATGTTCC3´

### Statistical analysis

In vitro assays were performed three times to better validate the experimental data. All the data were displayed as mean ± standard deviation (SD) using a one-way test by Minitab® 16.1.0 statistical software (Minitab Inc., State College, PA, USA). P values of less than or equal to 0.05 (one star), 0.01 (two stars), 0.005 (three stars) and 0.0001 (four stars) were considered as statistically significant.

## Results

### Characterization of cultivated dermal papilla cells and outer root sheet cells

**Fig.1:**
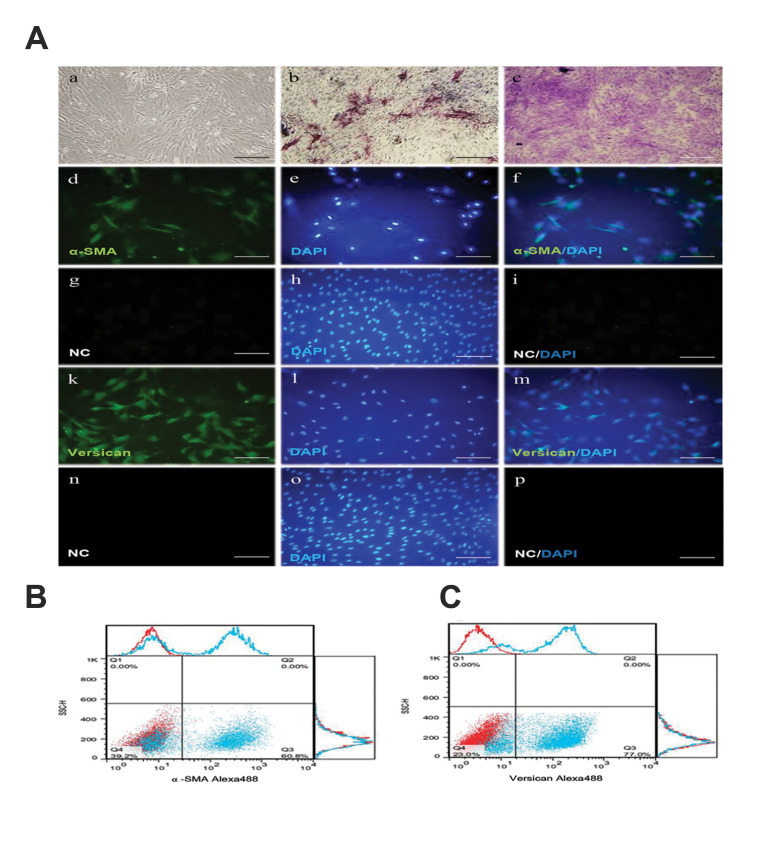
The morphology and cell profile markers of human dermal papilla cells (hDPCs). **A.** The morphology and cell profile markers of hDPC. **Aa.** hDPC showed a typical spindle-like morphology of fibroblast cell. **Ab.** Alkaline phosphatase activity (red) showed in hDPC passage two into a conventional culture medium. **Ac.** Dermal papilla colony tested strongly positive for toluidine blue staining (red). **Ad.** Immunofluorescent Imaging data was presented as α-SMA expression after culture (green). **Ae.** Nuclei were stained with DAPI (blue) and Af. Pseudo colored merged image. **Ag-Ai.** Secondary antibody control in hDPC. As a negative control, staining was performed without primary antibody, showing non-specific staining by the secondary antibody (Alexa Fluor 488 goat anti-mouse, (control). **Ak. **Immunofluorescent Imaging data was presented as versican expression after culture (green). **Al.** Nuclei were stained with DAPI (blue) and **Am. **Pseudo colored merged image. **An-Ap.** Secondary antibody control in hDPC. As a negative control, staining was performed without primary antibody, showing non-specific staining by the secondary antibody (Alexa Fluor 488 goat anti-rabbit, (control). Flow data are presented as dot plot and histograms from the specific markers (blue) overlaid with control (red) from hDPC. The dot plot and histogram flow cytometry analysis illustrated that hDPC were positive for **B. **α-SMA and **C. **Versican (scale bars: 200 μm, n=3).

As shown in figure 1Aa, the cultivated hDPCs grow in a spindle-shape with a sunflower morphology. The results of the ALP staining of the cultured hDPCs suggest these cells are able to induce hair growth (Fig .1Ab). Toluidine blue staining shows the potential for producing the extracellular matrix of hDPCs (Fig .1Ac). The results of immunostaining of the hDPCs showed the expression of α-SMA (Fig .1Ad-Af) and secondary antibody control in hDPC (Fig .1Ag-Ai), versican (green) and nuclei were stained with DAPI (blue, Fig .1Ak-Am) and secondary antibody control in hDPC (Fig .1An-Ap). The flow cytometry of the specific cytoplasmic markers of the DPCs showed expression of α-SMA (60.8%, Fig .1B) and versican (77%, Fig .1C).

**Fig.2:**
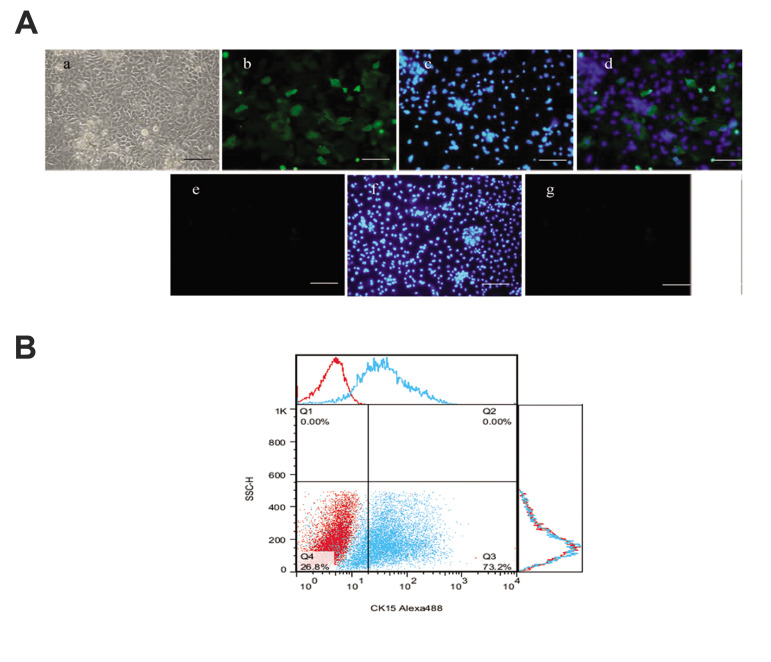
The morphology and cell profile markers of outer root sheet cells (ORSCs). **A. **The morphology and cell profile markers of ORSCs. **Aa. **ORSCs showed a typical morphology of polygonal-shaped structures. **Ab.** Immunofluorescent imaging data was presented as K15 expression after culture (green). **Ac.** Nuclei were stained with DAPI (blue) and **Ad.** Pseudo colored merged image. **Ae-g. **Secondary antibody control in human dermal papilla cells (hDPCs). As a negative control, staining was performed without primary antibody, showing non-specific staining by the secondary antibody [Alexa Fluor 488 Goat anti-mouse (control)]. **B. **Flow data is presented as histograms from specific markers (blue) overlaid with control (red) from ORSCs. The histograms of flow cytometry analysis illustrated that ORSCs were positive for K15 (scale bars: 200 μm, n=3).

The morphology of cultivated polygonal-shaped ORSCs is shown in Figure 2Aa. The results of the immunostaining of the ORSCs showed the expression of K15 (green) with nuclei stained with DAPI (blue, Fig .2Ab-Ad) and the secondary antibody control in hDPC (Fig .2Ae-Ag). The flow cytometry of the specific cytoplasmic markers of the ORSCs showed K15 (73.2%, Fig .2B). 

### Characterization of human adipose stem cells and platelet-rich plasma - exosomes

**Fig.3:**
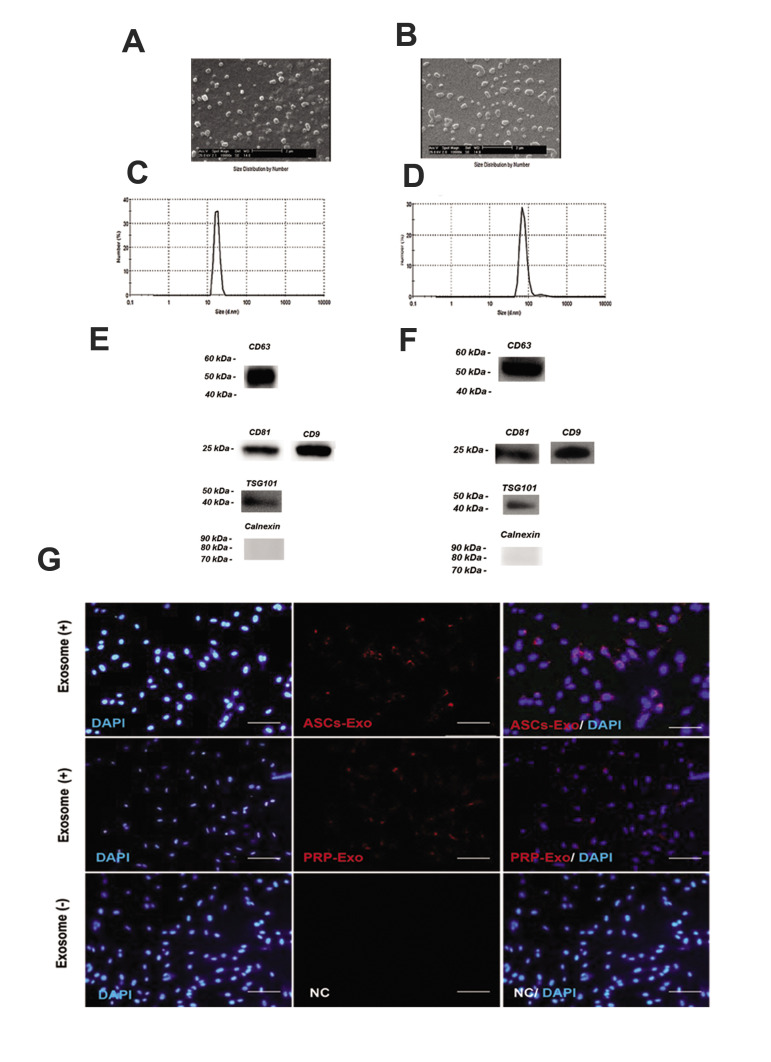
Characterization of exosomes derived from ADSc and PRP.** A, B.** SEM evaluation of exosomes (scale bars: 2 μm). **C, D. **Nanoparticle evaluation of ASC-Exo and PRP-Exo. The mean diameter of ASC-Exo and PRP-Exo were 167 and 147 nm. **E, F.** Immunoblotting for CD63, CD81, CD9, TSG101 and Calnexin in exosomes. **G. **Internalization of exosomes by human dermal papilla: confirmation of the uptake of exosomes in hDPC. The PKH26 (red) kit (20 μg/mL) were applied to stain ASC-Exo or PRP-Exo and incubated with hDPC for 24 hours (scale bars: 200 μm). PBS was used as negative control (NC). Nuclei were stained with DAPI (blue) for counterstaining (n=3). ADSc; Adipose-derived stem cells, PRP; Platelet-rich plasma, ASC; Adipose stromal cell exosome, hDPC; Human dermal papilla cell, and PBS; Phosphate-buffered saline.

The morphology of the exosomes was analyzed using electron microscopy after their isolation from the supernatants of human ASCs and cord blood PRP. According to the SEM results (Fig .3A, B), the round-shaped ASCs-Exo and PRP-Exo were 50-150 nm in diameter. The distribution and concentration of the exosomes were evaluated using NanoSight analysis. According to NanoSight Dynamic Light Scattering (Fig .3C, D), we found the majority of ASCs and PRP-Exo to be 30-150 nm in size. A few exosomal markers such as CD9, CD63, CD81, ITG101 and Calnexin were detected using western blotting (Fig .3E, F). The results of exosome characterization suggested the appropriate purity of specific exosomes.

### Internalization of exosomes by human dermal papilla

Previous research suggests that exosomes can enter different types of cells. The entrance of human ASCs and cord blood PRP exosomes into human DPCs was therefore examined. According to Figure 3G, the uptake of human ASCs and cord blood PRP exosomes by DPCs were evaluated using fluorescence microscopy. The results illustrated that exosomes can enter the DPCs cytoplasm and be localized in the perinuclear area. 

### ASCs-Exos and PRP-Exos induced dermal papilla migration, proliferation, and *in vitro* ALP activity

According to previous research, it is suggested that exosomes can promote the migration of DPCs. The results of the scratch closure test revealed ASCs-Exo group with concentration 100 µg/ml significantly increased migration of DPCs compared to experimental groups at 12- and 24-hour time points [Alexa Fluor 488 Goat anti-mouse (control)]. 

The MTS assay was used to evaluate the cell proliferation of DPCs at different concentrations of exosomes after 3 days (Fig .5). Investigating the metabolic activity of different sources of exosome through a two-dimensional culture of hDPCs showed a significant increase in 100µg/ ml of ASCs-Exo (Fig .5A) compared to PRP-Exo (Fig .5B).

### ASC-Exo induce *ALP, versican* and *α-SMA* expression in the cultured DPCs

According to (Fig .6), culturing the DPCs with ASC-Exo significantly increased the expression of *ALP, versican* and *α-SMA* compared to a co-culture with PRP-Exo. For 100 µg/ml of ASC-Exo, the expressions of ALP, versican and α-SMA proteins were respectively increased by a factor of 1.2, 2 and 3 compared to in the control group, suggesting the effectiveness of ASC-Exo in maintaining the trichogenic ability of DPCs. Although these results suggest that taking an exosome approach to the culture of DPCs can effectively maintain the trichogenic ability of these cells, further studies are required to obtain a clinically applicable protocol.

**Fig.4:**
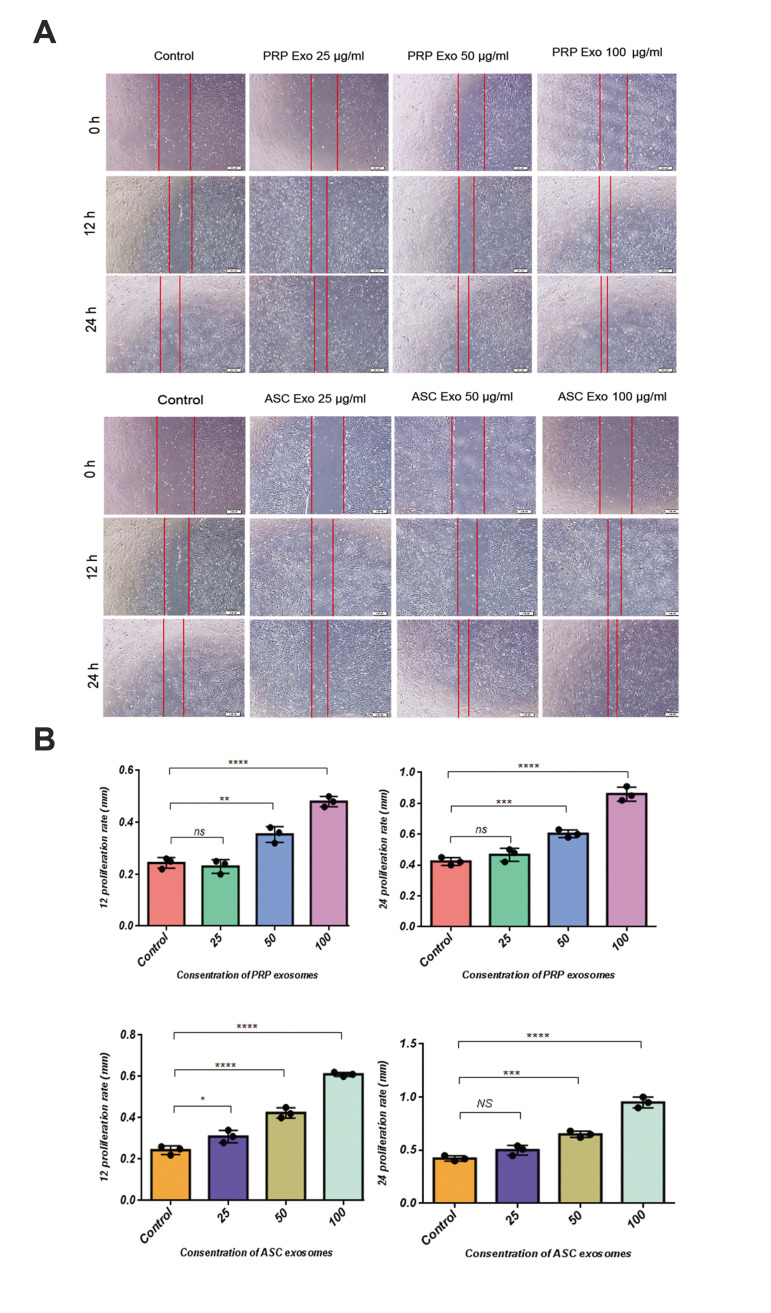
Scratch assay of human dermal papilla cells treated with different concentrations of ASCs and PRP exosomes. Relative wound area changes by different concentrations of **A.** ASCs exosomes and PRP exosomes treatment. ASCs-Exo or PRP-Exo were co-incubated with hDPC (left) and the scratch area at designated study points was normalized against that obtained at 0 hour. The cells cultured with serum (10%) were applied as control group. **B.** Light microscopy images of scratch assay of hDPC were manually delineated to ImageJ software analysis (scale bars: 200 μm, n=3). NS; Not significant, *; P<0.05, **; P<0.01, ***; P<0.005, ****; P<0.0001, ASCs; Adipose stromal cell exosome, PRP; Platelet-rich plasma, and hDPCs; Human dermal papilla cell.

**Fig.5:**
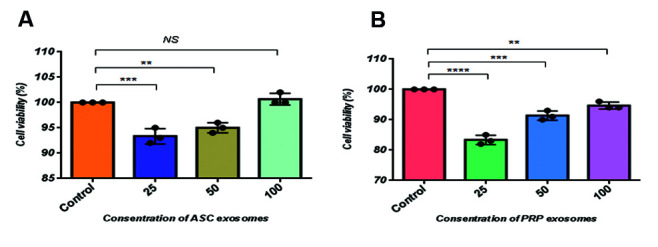
Cell proliferation and cell viability by MTS assay. Human DPC were plated into 96-well plates for 24 hours with or without different concentrations (25, 50, 100 mg/mL) of **A. **ASC-Exo and **B. **PRP-Exo. NS; Not significant, **; P<0.01, ***; P<0.005, ****; P<0.0001 (n=3), DPC; Dermal papilla cell, ASC; Adipose stromal cell exosome, and PRP; Plateletrich plasma.

**Fig.6:**
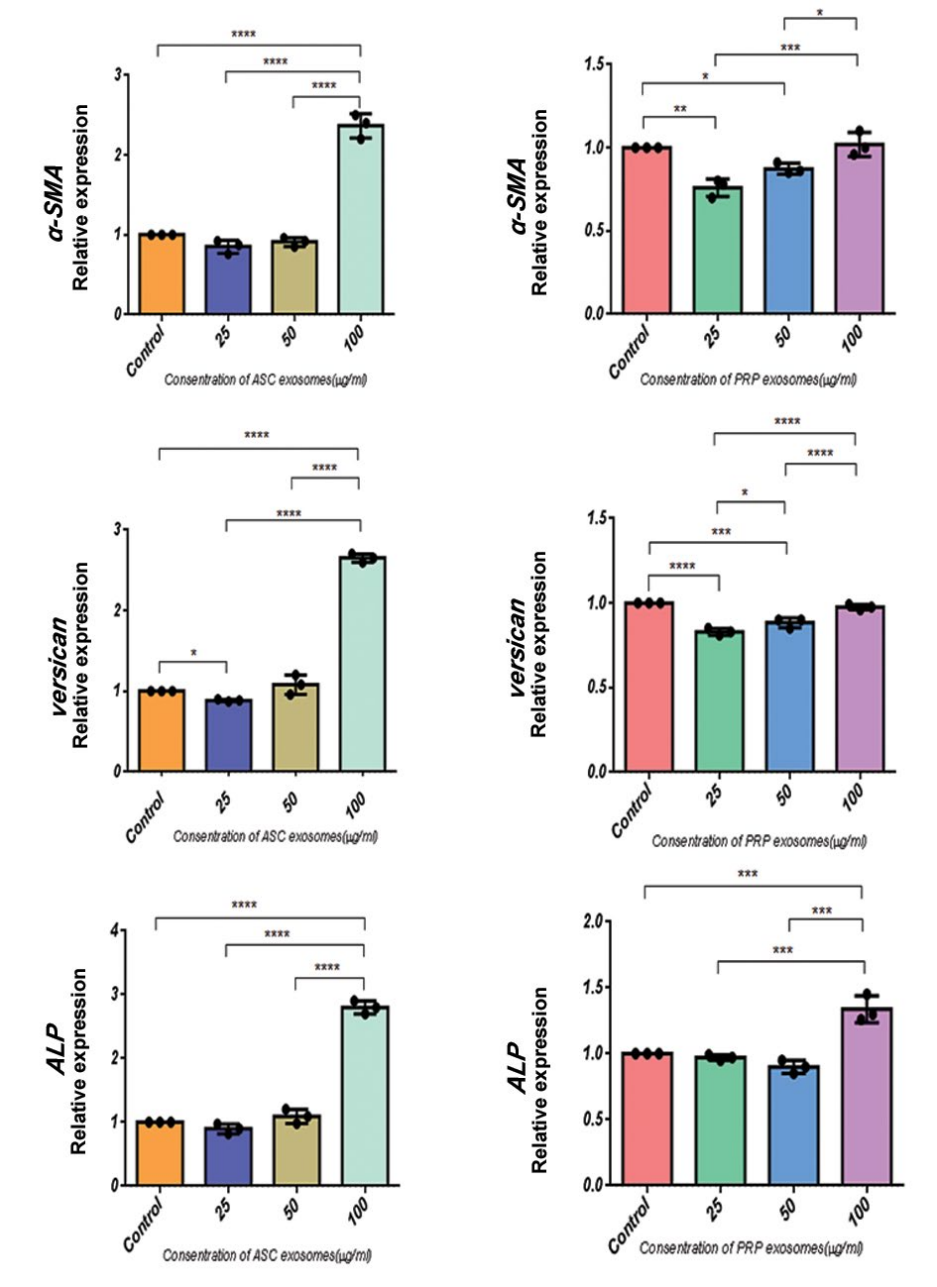
Comparison of the *α-SMA, versican* and *ALP* mRNA expressions following different concentrations of ASCs and PRP exosome treatments. Different experimental groups such as 100, 50 and 25 μg/mL of ASC-Exo or PRP-Exo were incubated with hDPC for 48 hours respectively. The qRT-PCR analysis and the expression of each gene of exosome-treated hDPC was normalized against the expression of cultivated cells with serum (10%), which were used as the control group. All data are expressed as mean ± standard deviation (SD) from three replicates. *; P<0.05, **; P<0.01, ***; P<0.005, and ****; P<0.0001 (n=3).

## Discussion

DPCs play a key role in regulating the behavior of epithelial cells in terms of morphogenesis and function in hair follicles (14, 15). Hair growth can be maintained and new hair formation may be induced by DPC singling and growth factors (16, 17). Transplanting a combination of DPCs and keratinocytes in nude mice was found to induce hair follicle formation. Losing the hair follicle inductivity after several passages in tissue culture and limited hair follicle sampling were, however, the major obstacles in transplantation of DPCs (18-20). Therefore, we examined the possibility of a new method to maintain hair follicle inductivity of dermal papilla cells in a 2D cell culture condition. Our result indicated that a combination of ASC secretory growth factors in DP cell culture maintain hair follicle inductivity after several passages compared to conventional methods. 

Recent studies suggest that paracrine mechanisms such as conditioned medium and exosomes play a major role in cell-based therapies (21). ADSC-derived secretory growth factors, such as keratinocyte growth factor, vascular endothelial growth factor (VEGF), platelet-derived growth factor (PDGF) and hepatocyte growth factor are effective in hair regeneration. The paracrine mechanism of an ADSC-conditioned medium was shown to be improved by hypoxia (22). In addition, PRP contains different growth factors, such as PDGF, transforming growth factor β (TGF-β), VEGF, epidermal growth factor (EGF), insulin-like growth factor (IGF) and fibroblast growth factor (FGF), which have been applied to treat androgenic alopecia and chronic wounds (23, 24). Despite the promising results of using stem cell-derived conditioned media (CM) in treating different diseases, the short half-life of cytokines and growth factors present in CM is a disadvantage for regenerative therapy (25).

In the field of dermatology, exosomes have been found to have an effective role in wound healing, preventing scarring and inducing hair growth. Exosomes can therefore provide more efficient and safer treatments compared to CM and stem cell therapy (26, 27). Different components of exosomes, such as RNAs, miRNAs and proteins mediate cell-cell communication and function. Protection from degradation is a specific advantage of exosomal therapeutic applications compared to CM (21). The other advantages include long term shelf life, long-range of intracellular communications, simple storage and lower risks of immune system reactions following treatment (28). The results of previous studies have shown that activation of Erk and Akt signaling pathways along with PRP-Exo induce cell angiogenesis (29). PRP-Exo can increase the proliferation and migration of endothelial cells and fibroblasts in chronic wounds (30). Therefore, in this study two groups of exosomes were used to evaluate the maintenance of thricogenic ability of dermal papilla in a 2D cell culture condition. Our results revealed that the culture of human DPCs with ASC-Exo exhibited significantly increased migration, proliferation and hair inductivity compared to other experimental groups. In the hair cycle, MSC exosomes can stimulate the progression of the hair follicle telogen phase to the anagen phase (31, 32). A previous study has found activation of Wnt/β-catenin signaling by umbilical cord MSC-derived exosomes in a site of wound healing (33). Other recent findings have shown that DPC exosomes can delay the conversion the hair anagen phase to the catagen phase through activation of β-catenin and Shh signaling pathways (32). 

Thus, our results are consistent with the other reports (25, 31, 32) that exosomes maintain *in vitro* hair inductivity of dermal papilla cells. The results of our study demonstrated that when 100 µg/ml ASCs-Exo was compared to the same concentration of PRP-Exo, the former significantly promoted DP proliferation and migration, as well as expression of ALP, versican and α-SMA proteins. Thus, ASCs-Exo provide a novel effective approach for the treatment of hair loss. Although the mechanism of exosomes is still unclear, we recommend the exact mechanism of exosomes to investigate this issue due to their rich combination of different RNAs, growth factors and proteins which are dependent on the function of adipose derived mesenchymal cells.

## Conclusion

We successfully applied exosomes as a new method to support hair inductivity of human DPCs and to improve the outcome of hair loss treatment. Using a different strategy for the maintenance of hair inductive capacity of dermal papilla cells may provide an appropriate method for discovering new therapeutic goals for hair regeneration. Further studies should not only aim to use a different strategy with different compounds and adjuvants but also expand the existing knowledge on the maintenance of dermal papilla hair inductivity.
